# A delirium prevalence audit and a pre and post evaluation of an interprofessional education intervention to increase staff knowledge about delirium in older adults

**DOI:** 10.1186/s12912-021-00692-2

**Published:** 2021-10-19

**Authors:** Beverley Ewens, Karla Seaman, Lisa Whitehead, Amanda Towell-Barnard, Michelle Young

**Affiliations:** 1 School of Nursing and Midwifery, Edith Cowan University, 270 Joondalup Drive, Joondalup, WA 6027 Australia; 2 Joondalup Health Campus, Cnr Grand Boulevard and Shenton Avenue, Joondalup, WA 6027 Australia

**Keywords:** Delirium, Delirium knowledge, Symptom recognition, Delirium assessment, Interprofessional education, quality improvement

## Abstract

**Background:**

Delirium is more prevalent in older people and estimated to occur in up to 50% of the hospital population. Delirium comprises a spectrum of behaviours, including cognitive and attention deficits, and fluctuating levels of consciousness, often associated with an underlying physiological disturbance. Delirium has been increasingly associated with adverse outcomes. Although often preventable or can at least be mitigated, delirium may not be a standard part of assessment and thus may not be recognized in the early stages when it is most likely to be treated successfully. The aim of this study was to evaluate the level of knowledge of delirium amongst clinicians caring for patients at high risk of developing delirium and to determine whether education can improve clinical assessment of delirium.

**Methods:**

Two hundred and forty-six case notes were audited before and 149 were reviewed after the education intervention and implementation of a delirium screening tool. Clinicians at the hospital were invited to complete a questionnaire on knowledge of delirium. The questionnaire was based on a validated tool which contained 39 questions about delirium. The questionnaire also contained 28 questions on delirium knowledge. Additional questions were included to gather demographic information specific to the hospital. Descriptive statistics, chi square and independent t-tests were conducted to test for differences in knowledge between the pre and post periods. The Squire Checklist Reporting Guidelines for Quality Improvement Studies informed the preparation of the manuscript.

**Results:**

The audit demonstrated that the use of a cognitive assessment tool overall increased from 8.5% in pre education to 43% in the post education period. One hundred and fifty-nine staff completed the questionnaire in total, 118 the pre and 41 post. The knowledge subscale score was high pre and post education and no statistically significant difference was observed. The greatest increase in knowledge was related to knowledge of the risk factors subscale. The increase in knowledge (6.8%) was statistically significant.

**Conclusion:**

An interprofessional approach to delirium education was effective in not only increasing awareness of the factors associated with this syndrome but also increased the use of a delirium assessment tool.

**Supplementary Information:**

The online version contains supplementary material available at 10.1186/s12912-021-00692-2.

## Introduction

Acute onset delirium is a distressing condition for both patients and their families and is becoming a significant issue within health care. In recent years delirium has been increasingly associated with adverse outcomes [1], especially in older people [2, 3]. These adverse outcomes include extended length of hospital stay [4], cognitive decline [5], increased re-admission rates [6], increased health care costs [6–8], functional decline and mortality [9, 10]. It is more prevalent in the older population and comprises a spectrum of behaviors, including cognitive and attention deficits and fluctuating levels of consciousness [11, 12] and can frequently be prevented or mitigated [13]. Delirium is often associated with an underlying physiological disturbance [14], is multifaceted and estimated to occur in up to 50% of the older hospital population [15] and up to 80% of the intensive care population [16]. Risk factors for delirium include a previous history of delirium, dementia or cognitive impairment pre-hospitalisation [17]. Acute delirium can be precipitated by many causes including acute alcohol withdrawal [18], sepsis [19] and some medications [20]. A diagnosis of delirium not only yields costs to the individual and their families but also financial implications to organisations [7], and yet it has been estimated that up to 40% of cases could be prevented with appropriate management and early intervention (Johansson, Bergh, Ericsson, & Sarenmalm, 2018).

The aim of this study was two-fold. Firstly, to evaluate the level of knowledge of delirium amongst clinicians caring for patients at high risk of developing it. Secondly to determine whether an interprofessional education program could improve clinical assessment of delirium in high risk patients.

## Background

It is irrefutable that clinical staff play a vital role in the recognition and management of delirium [21]. Yet a lack of knowledge of the risk factors associated with delirium and the recognition of symptoms of delirium by nursing and medical staff, have been identified as contributing to under assessment and potentially inappropriate management (Buettel, Cleary, & Bramble, [22]; Jenkin et al., [23]; Sinvani, Kozikowski, Pekmezaris, Akerman, & Wolf-Klein, [24]). To be able to increase recognition and appropriate management it is therefore imperative that staff have the knowledge to recognise the presentation and can initiate the management of delirium and mitigate the sequelae of events which can ensue [25]. Knowledge of delirium and the ability to recognise its manifestations is only one component of the issue. The management of delirium is challenging and can vary. Predominantly management currently focusses on pharmacological and non-pharmacological methods including antipsychotics which are much more controversial [26, 27]. This controversy and lack of consensus around best practice in relation to the management of delirium presents challenges to researchers and clinicians [28].

Education on the recognition and management of delirium is widely provided within undergraduate nursing curricula in various international contexts including The United States [29], Northern Ireland [21], undergraduate medical curricula in the UK ([30–32] as well as in undergraduate interprofessional learning contexts [33]. Yet this knowledge developed at undergraduate level does not always transfer to professional practice as under recognition of delirium in clinical contexts persists [34, 35].

When dedicated delirium education is provided for clinical staff, it has demonstrated an increase in knowledge and associated prevention of delirium and promoted early detection and appropriate management [25, 36, 37].

In addition to education to support the recognition of delirium in practice, validated delirium assessment tools have been developed and widely implemented to enable clinicians to assess patients accurately and implement appropriate management strategies based on objective measures [38]. Twenty-one of those assessment tools have been identified for use within clinical practice [39]. The most frequently used tools are The Confusion Assessment Method (CAM) [40], CAM-ICU [41], The Delirium Rating Scale (DRS) [42] and the DRS revised version DRS-R98 [43]. The 4AT [44] utilised in this study, is most suited to the acute care population, takes 2 min to complete and does not require special training to learn [45]. The application of delirium assessment tools and the identification of delirium have been identified as facilitating the delivery of appropriate management [46] and therefore reducing associated morbidity [47]. Selecting an appropriate delirium assessment tool has been identified as the first stage in improving assessment and management of patients with delirium as well as increasing knowledge and education about it [45].

Oh et al. [28] acknowledged that there are significant issues in health around the definition and assessment of delirium. Oh et al. [28] consider there to be a lack of diagnostic testing, a lack of accepted definitions and inadequate consensus on the assessment methods, all of which complicate the research into and management of this complex syndrome. The development, validation and standardization of assessment tools have been called for to prevent the existing disparity in delirium assessment [28].

## Methods

### Design

The first stage of the project was an audit of the prevalence of delirium assessment on an acute admission ward. The second stage of the project was the implementation of a delirium assessment tool following a staff education program. The third stage was a review of delirium assessment rates and staff knowledge pre and post the interprofessional education (IPE) intervention. The Squire Checklist Reporting Guidelines for Quality Improvement Studies informed the preparation of the manuscript [48].

### Participants

A convenience sample of clinical staff across 12 wards, at a large general hospital in metropolitan Western Australia, were invited to participate in the study. This included nurses (registered and enrolled), allied health, and medical (registrars and consultants) staff.

### Setting

The hospital has 722 beds, 73,000 inpatients per year and approximately 3000 clinical staff. The prequestionnaire was conducted in November 2017 and the post questionnaire in January 2018. The clinical audit was conducted in April 2017 and repeated in May 2018.

### Delirium prevalence audit

Prior to the implementation of a delirium assessment tool, there were no specific delirium assessment tools in place at the study site. The diagnosis of delirium was identified within the audit if cognitive impairment was suspected and validated tools to measure cognitive impairment were used in the assessment process including the Standardized Mini-Mental State Examination [49] or the Montreal Cognitive Assessment (MoCA) [50]. In addition to this assessment, a clinical assessment and diagnosis of delirium was confirmed by medical practitioners and recorded in the patients’ notes. In April 2017, prior to the trial of the delirium screening tool, a total of 246 patient records were reviewed and in May 2018, 6 months post implementation of the tool, 149 records were reviewed for the prevalence of delirium. The medical records of all patients aged over 65 years admitted to an acute admission ward were selected for an entire month for auditing. In 2017, prior to the implementation of the education intervention and delirium assessment tool, patients’ medical records were audited for documented evidence of an assessment for cognitive impairment being undertaken on admission and/or anytime during the hospital stay. This assessment was undertaken by nursing and/or medical staff using validated cognitive impairment assessment tools which comprised The Standardised Mini-Mental State Examination (SMMSE) [49] and the Montreal Cognitive Assessment (MoCA) [50]. The second audit in 2018 was post the education intervention and implementation of the delirium assessment tool and measured the use of this tool within the same ward.

### Delirium screening tool

The hospital introduced a delirium screening tool, the 4AT [44] in November 2017, designed to be used to screen all adult patients over the age of 65 years for acute delirium, who were admitted to the emergency department, Medical Assessment Unit or surgical wards. The screening tool was adapted by members of the research team at the study site to include a delirium management algorithm. The algorithm was developed from best available evidence and guided the clinicians in the most appropriate management of patients diagnosed with acute delirium. However, the assessment component of the tool remained unchanged from the validated version.

The education intervention was developed and implemented at the study site over a 1 month period in December 2017. The education comprised a freely available training video which detailed the prevalence, predisposing factors, differing presentations and associated morbidity and mortality of delirium. The video was followed by targeted small group education sessions in ward areas, delivered by the hospital’s nursing and medical educators. Education was also delivered at grand rounds and meetings of the hospital’s clinical leadership team. These sessions were designed to enable interprofessional group discussion about delirium as a syndrome, the screening tool and the fundamental concepts of delirium presentation and management.

### Questionnaire design

The delirium knowledge questionnaire is a validated tool developed by Landsborough et al. [51]. The original questionnaire contained 36 closed questions plus a demographic section. The demographic section was adjusted to the hospital’s staff profile and included information on age, gender, role, length of time in current position, working hours per fortnight, number of years in profession and the main ward area of work. The first section asked about the definition of delirium and comprised four multiple-choice questions, this was followed by a list of seven delirium assessment scales and a request to identify which scales should be used to assess for delirium, dementia, depression or none. An additional three tools were added to this list as they were commonly used within the hospital. The remaining 28 from a total of 36 questions comprised statements that required responses of agree, disagree or unsure. The questions could be broken down into two subscales consisting of 14 questions each; one subscale was on the general knowledge of delirium and the other on the risk factors associated with it.

### Data analysis

The data from the delirium prevalence audit were analysed utilising descriptive statistical methods. The prevalence of delirium was 6.5% in 2017 and 4.7% in 2018. Questionnaire data were downloaded from the electronic questionnaire instrument Qualtrics if completed electronically or entered into a case report form if paper based. Data analysis was completed using STATA Version 15. The questionnaires were marked to calculate a total score plus two subscale scores. Descriptive statistics were used to summarise the demographic data and scores. Differences in demographic data and individual questions were analysed with chi square. Difference in total and subscale scores were analysed using independent t-tests. A *p* value less than 0.05 was taken as statistically significant.

A post-hoc analysis measuring the effect size for the mean difference in the total mean score produced a Cohen’s d of − 0.44 (95%CI: − 0.80—0.08), this is a medium effect size. The power of this study calculated using G*Power was 0.67. Hence, the discrepancy in the numbers of participants in the pre and post questionnaire is a limitation. There is an increased risk of a TYPE II error where the accepted standard of 0.8 has not been met.

For future research it is suggested to aim for a sample size of 83 in the pre and post to ensure an adequate power of 0.8. Due to the limited number of delirium cases identified across the audit sample only descriptive results have been provided. Missing data were not included in the analysis.

## Results

### Audit

The prevalence of delirium in the clinical records audited was 6.5% in 2017 and 4.7% in 2018. The audit demonstrated that the use of a cognitive assessment tool in this cohort of patients, increased from 8.5% (*n* = 21) in 2017 to 43% (*n* = 64) in 2018, with most staff now using the cognitive impairment/delirium screening tool where indicated.

Overall, in 2018 compared to 2017, the patients admitted to the hospital were younger in age (mean age), a higher percentage were males (5%), were 6% less likely to have a length of stay greater than 10 days and 4% less had a previous history of dementia (See Supplementary Table D for full descriptive statistics of the audit findings by year).

### Questionnaire

In total, 118 staff completed the pre-education questionnaire and 41 completed the post education questionnaire. The demographic characteristics are outlined in Table 1. The demographics of the staff completing the pre and post questionnaire were similar. Most respondents were female.

**Table 1:** Demographic characteristics of staff undertaking the education intervention

Demographics	Pre (***n*** = 118)	Post (***n*** = 41)
**Age, n (%)**		
21–30	33(27.9)	15 (37.5)
31–40	32 (27.1)	8 (20.0)
41–50	26 (22.1)	6 (16.0)
51–60	27 (22.9)	11 (27.5)
**Gender, n (%)**		
Male	9 (7.6)	6 (14.6)
Female	109 (92.4)	35 (85.4)
**Role, n (%)**		
Nurse	81 (68.6)	29 (70.7)
Medical	4 (3.4)	4 (9.8)
Allied Health	33 (28.0)	8 (19.5)
**Length of time in current position, n (%)**		
Less than 6 months	14 (11.9)	7 (17.1)
6 to 12 months	10 (8.5)	4 (9.7)
More than 12 months	94 (79.6)	30 (73.2)
Other		
**Working hours per fortnight, n (%)**		
Less than 40	14 (12.0)	2 (4.9)
40 to 64	46 (39.3)	19 (46.3)
More than 64	57 (48.7)	20 (48.8)
**Number of years in current profession, n (%)**		
5 or less	44 (37.6)	18 (45.0)
6 to 12	36 (30.8)	12 (30.0)
13 to 20	16 (13.7)	5 (12.5)
More than 20	21 (17.9)	5 (12.5)
**Main Ward/Area, n (%)**		
Medical	49 (41.5)	15 (36.6)
Rehabilitation	33 (28.0)	14 (34.1)
Surgical	36 (30.5)	12 (29.3)

For both the pre and post questionnaire, nine in ten respondents answered the delirium definition questions correctly (chi squared test; *p* = 0.948). Respondents’ knowledge of which tool should be used to assess for specific conditions varied. There was a statistically significant increase in awareness of the appropriate use of the Montreal Cognitive Assessment (MoCA) [50] for dementia, 84 (74.3%) pre education, and 37 (92.5%) post education (*p* = 0.015) (See Supplementary Material Table A for staff knowledge about the different rating scales commonly used).

There was a significant increase in the combined knowledge and risk subscales’ total score before and after the intervention and audit. The knowledge score remained high in both the pre and post education intervention, with no statistically significant difference between the two scores. The largest increase was observed in the risk subscale, which was statistically significant (see Table 2).

**Table 2:** Mean percent score pre and post for the total survey and two subscales

	Pre Mean Percent (%)	Post Mean Percent (%)	Difference	***P*** value*
**Total Score**	63.0	68.4	5.4	**0.016**
**Knowledge Subscale**	77.1	81.0	3.9	0.1448
**Risk Subscale**	48.9	55.7	6.8	**0.014**

The Pearson correlation coefficients amongst the knowledge and risk subscales was 0.30 (*p* < 0.01) in the pre and 0.4312 (*p* < 0.01) in the post questionnaire, demonstrating that there were medium to moderate associations between the two subscales that is a statistically significant relationship for both the pre and post questionnaire.

There was no difference in the percentage of correct answers for the knowledge subscale in the post questionnaire compared to the pre. The percentage of correct answers ranged from 56.1 to 97.6% in the knowledge subscale questions (See supplementary Table B). Whilst the risk subscale demonstrated that for two questions, there was an increase in the number of correct responses in the post questionnaire compared to the pre. Participants correctly responded ‘false’ to the statements “Gender has no effect on the development of delirium” (*p* = 0.006,), and “A family history of dementia predisposed a patient to delirium” (*p* = 0.001). The percentage of correct answers ranged from 7.6 to 97.5%. (See Supplementary material Table C).

Nurses demonstrated a significant increase in delirium knowledge, with a pre-intervention mean score of 60.8% and a post-intervention score of 65.4% (*P* = 0.045). Due to the small sample of medical and allied health professionals, no statistical tests were conducted.

## Discussion

The prevalence of delirium reported in this study was less than has been reported elsewhere in similar contexts [8, 52]. This may be due to the prevalence of risk factors within our study population, but this can only be postulated. A high prevalence of delirium has been identified across different subsets of patients which were not present in our study including radiotherapy, visceral surgery, reconstructive plastic surgery, cranio-maxillofacial surgery [8], intensive care [53] and trauma [54].

The use of a delirium assessment tool also increased during this study within the same patient population. Delirium assessment in high risk patients has been identified as particularly important in the recognition of subtypes, particularly hypoactive type, which is the least recognised and appropriately managed [55].

Participants’ knowledge of delirium pre and post the education intervention did not reach statistical significance, with levels of knowledge already high pre-education. Theoretical knowledge of delirium by nurses has also been reported elsewhere as high [56], although this study did identify that theoretical knowledge did not necessarily translate into practice. The reasons why theoretical knowledge may not transfer to practice has not been explored in the literature or how this translation to practice can be enhanced through modes of education and is worthy of further exploration. In a study with a cohort of junior doctors, although participants recognised the clinical significance of delirium they demonstrated poor knowledge of diagnostic criteria [57]. Contributing factors to this finding were lack of training and perceived disinterest from senior colleagues. Baseline knowledge of delirium has been shown to be higher in the most experienced nurses [58] and those with baccalaureate and master’s degrees [59, 60], however, we did not explore the relationship between level of knowledge, clinical experience or qualifications in our study.

Specific areas of lack of knowledge in our study included recognition of delirium, predisposing factors and the medications which can precipitate delirium. Delirium cannot be managed and the potentially catastrophic consequences of it mitigated, unless clinicians involved in direct care are able to recognise it. Families of patients may be able to play a significant role in alerting staff to abnormal behavioural changes in their family members. Family initiated escalation of care has been explored in other contexts [61, 62] and a family centred approach to delirium recognition and management should be further explored. In our study the largest increase post education intervention which reached statistical significance in this study was observed in the risk subscale. Lack of knowledge of risk factors has also been identified elsewhere within a cohort of nurses and physicians [63]. Similar findings were reported in another study of nurses undergoing an e-learning delirium education program [60], with increases in knowledge in this area post education intervention reaching statistical significance. This lack of knowledge surrounding risk factors, as in this study, may impact on the recognition and management of delirium. It is acknowledged that all patients are at risk of developing delirium during their illness journey [7], it is therefore recommended that risk factors for delirium be identified during the admission assessment process for all patients, so that staff may be particularly alert to patients at risk of developing it.

In this study, delirium assessment was undertaken on all patients over 65 years, but it is important that clinicians are aware that delirium can manifest in younger people who have identified risk factors, particularly those who have had an ICU experience [64]. This should be an essential component of delirium education programs to enable clinicians to identify those at risk of delirium and instigate interventions which may mitigate the onset, rather than treating delirium when it manifests.

An IPE strategy in this study was an effective approach in increasing the recognition of delirium symptoms, assessment and management in this small cohort of participants. Interprofessional education strategies in other contexts have been reported to increase interprofessional communication, collaboration, patient communication and understanding of carer roles [65]. In a study with orthopaedic nurses where knowledge of delirium was measured post an education intervention based on a national education program, baseline knowledge significantly improved following the education intervention [59]. In a systematic review [66], the application of interactive and authentic instructional methods supported by widely available guidelines and resources, case study discussions as well as the appointment of dedicated delirium clinicians, were all effective interventions in the recognition and management of delirium [66]. The studies within this review included education interventions which were site specific and developed in-house, however, this does suggest that the interactivity of educational resources, supported by authentic content and evidenced based guidelines is an effective educational approach. Despite the effectiveness of educational interventions the effect on the level of knowledge has been recognised to decline over time [67], indicating that regular education updates are important to maintain the currency of knowledge in this area.

Participants’ knowledge of the use of a variety of assessment tools improved post education intervention, with an increased knowledge of the application of MoCA reaching statistical significance. This may be because staff who responded were from surgical and acute medical areas where MoCA may not be frequently used and which could account for the increase in knowledge post education intervention. All other tools included in the education would be used throughout all areas of the site and therefore already familiar to the participants.

This study did not capture staff knowledge of the recognition of delirium superimposed on a diagnosis of dementia, which has been identified in the literature as an issue for nurses [68]. Potentially this could also add to under recognition of delirium and is worthy of exploration at the study site.

Medical science is advancing at an exponential rate and as such clinicians should be cognisant that emerging treatments and practices may also precipitate delirium. There is currently no recognised, widely available interprofessional and evidence-based education program for improving delirium knowledge, practice and management. Any education initiatives currently available appear to be locally developed and implemented in a non-standardised approach. This could lead to many inconsistencies including quality of the program, the application of appropriate pedagogical approaches, evidence-based content and ultimately in the management of patients, even within individual sites.

### Strengths of the study

This study illustrated that a focused IPE approach increased the use of a delirium screening tool at the study site and also improved staff knowledge of delirium. This study has also shown how a collaborative approach between clinicians and academics provides an opportunity for education interventions which have the potential to improve practice and ultimately impact on patient care.

### Limitations

Limitations of this study should be acknowledged. The discrepancy in the completion rate pre and post the education intervention is a significant limitation. The length of time between the pre and post measures determined that it is not possible to confirm if the increase in knowledge demonstrated post the education intervention was due to the education intervention itself, or the synergistic effect of the implementation of the screening tool and the education intervention. The limitations of a retrospective case note review as the audit method is also acknowledged as the team were reliant on accurate record keeping at the time the entries were made. We did not record how many of the staff who undertook the education intervention, worked on the ward where the delirium prevalence audit took place and which could have influenced the findings of the audit. The study was underpowered which must be taken into account, where large differences in the results could be due to lack of power to be able to identify statistical difference. The significant differences between the pre and post education sample has limited the interpretation of the data. Although the audit tool was piloted, intra and inter-rater reliability of the tool were not established which may have affected the reliability and validity of the data. Delirium assessment tools were not a component of usual practice at the site before the implementation of the delirium assessment tool. It cannot be determined if the increase in the use of the delirium assessment tool was due to the education or requirement of the use of the tool in all patients over the age of 65 years. The SMME in the validated knowledge questionnaire was defined as a delirium assessment tool which it is not. This is also a limitation of this study. The study was undertaken in a tertiary hospital setting and the relevance of the findings to other settings is unknown.

## Conclusion

Acute delirium can yield significant financial and human costs for those affected by it. Delirium is still widely under recognised and managed within health care, and there is a pressing need to increase the recognition of delirium itself but also of the predisposing factors. This study identified that an IPE approach to delirium education did increase overall knowledge of delirium, particularly in the nursing cohort, but even though the recognition of risk factors increased, they remained poorly recognised. At present, there are insufficient education programs on delirium in hospitals, even though evidence shows it goes unrecognized all too often. Making this education available to hospital staff regularly could improve the recognition and therefore evidence-based management of this clinical problem. Including such education in annual clinical staff mandatory updates staff could have a significant impact on the identification and management of delirium. As the multidisciplinary team is responsible for the care of the patient with delirium, an IPE approach to this education is an obvious one and should be adapted to include recognition of delirium, associated risk factors and evidence-based management. This education should be based on research informed education strategies including simulation and the use of case studies as well as validated assessment methods. This study did not explore the recognition of delirium in all contexts including when superimposed on dementia. This may be an area of further exploration to be included in education initiatives.

### Implications for clinical practice

An IPE approach can be an effective way to increase knowledge of delirium and its management as well as potentially standardising practice. The methods used in this education intervention have potential to be adapted to other contexts across this particular site.

## Supplementary Information

**Additional file 1: Table A**. Results for rating scales commonly used to detect certain conditions for cognitive impairment, delirium, dementia, _depression or none._ *Chi square test

**Additional file 2: Table B.** Results for questions relating to knowledge of delirium

**Additional file 3: Table C.** Results for questions relating to risk of delirium

**Additional file 4: Table D**. Descriptive statistics of audit findings by year. *health record

## Data Availability

The datasets used and/or analysed during the current study are available from the corresponding author on reasonable request.
