# Supplementary material for: A delirium prevalence audit and a pre and post evaluation of an interprofessional education intervention to increase staff knowledge about delirium in older adults
Source: BMC Nurs. 2021 Oct 19;20:202. doi: 10.1186/s12912-021-00692-2 (PMC8525041; doi:10.1186/s12912-021-00692-2)
Supplement: Supplementary file 1 — Additional file 1: Table A. Results for rating scales commonly used to detect certain conditions for cognitive impairment, delirium, dementia, depression or none. *Chi square test [file 12912_2021_692_MOESM1_ESM.docx]

Table A. Results for rating scales commonly used to detect certain conditions for cognitive impairment, delirium, dementia, depression or none

| **Rating Scale/Tools** | **Conditions** | **Pre - Correct**  **responses, n**  **(%)** | **Post -** | **P value*** |
| --- | --- | --- | --- | --- |
|  | **used for** |  | **Correct** |  |
|  |  |  | **responses, n** |  |
|  |  |  | **(%)** |  |
| **Mini Mental State** | Cognitive impairment &  de | 16 (13.9) | 2 (4.9) | 0.120 |
| **Examination (MMSE)** | dementia |  |  |  |
| **Glasgow Coma Scale (GCS)** | None | 64 (56.6) | 22 (57.9) | 0.892 |
| **Delirium Rating Scale (DRS)** | Delirium | 108 (93.9) | 40 (97.6) | 0.363 |
| **Alcohol Withdrawal Scale** | Delirium & | 94 (81.0) | 37 (92.5) | 0.088 |
| **(AWS)** | None |  |  |  |
| **Confusion Assessment** | Delirium | 49 (41.9) | 21 (52.5) | 0.243 |
| **Method (CAM)** |  |  |  |  |
| **Beck’s Depression Inventory** | Depression | 104 (90.4) | 39 (97.5) | 0.150 |
| **Braden Scale** | None | 106 (91.4) | 38 (92.7) | 0.795 |
| **4AT** | Delirium | 36 (36.0) | 16 (47.1) | 0.253 |
| **Montreal Cognitive** | Cognitive impairment Dementia | 84 (74.3) | 37 (92.5) | **0.015** |
| **Assessment (MoCA)** |  |  |  |  |
| **Abbreviated Mental Test** | Dementia | 50 (43.9) | 22 (55.0) | 0.224 |
| **(AMT)** |  |  |  |  |

*Chi square test
