# Supplementary material for: A delirium prevalence audit and a pre and post evaluation of an interprofessional education intervention to increase staff knowledge about delirium in older adults
Source: BMC Nurs. 2021 Oct 19;20:202. doi: 10.1186/s12912-021-00692-2 (PMC8525041; doi:10.1186/s12912-021-00692-2)
Supplement: Supplementary file 2 — Additional file 2: Table B. Results for questions relating to knowledge of delirium [file 12912_2021_692_MOESM2_ESM.docx]

Table B. Results for questions relating to knowledge of delirium

| Questions | Pre - Correct  Answer n (%) | Post - Correct  answer n (%) | P-value |
| --- | --- | --- | --- |
| Fluctuation between orientation and disorientation is  not typical of delirium (False) | 89 (76.1) | 36 (87.8) | 0.112 |
| Symptoms of depression may mimic delirium (True) | 54 (46.2) | 19 (46.3) | 0.983 |
| Treatment of delirium always includes sedation (False) | 110 (93.2) | 40 (97.6) | 0.300 |
| Patients never remember episodes of delirium (False) | 79 (68.1) | 33 (82.5) | 0.081 |
| A Mini Mental Status Examination (MMSE) is the best  way to diagnose delirium (False) | 69 (58.5) | 30 (73.2) | 0.094 |
| Delirium never lasts for more than a few hours (False) | 110 (94.0) | 39 (95.1) | 0.793 |
| A patient who is lethargic and difficult to rouse does not  have delirium (False) | 80 (68.4) | 23 (56.1) | 0.156 |
| Patients with delirium are always physical and/or  verbally aggressive (False) | 112 (94.9) | 39 (95.1) | 0.958 |
| Delirium is generally caused by alcohol withdrawal  (False) | 100 (84.8) | 39 (95.1) | 0.084 |
| Patients with delirium have a higher mortality rate  (True) | 67 (56.8) | 23 (56.1) | 0.939 |
| Behavioural changes in the course of the day are typical  of delirium (True) | 83 (70.3) | 30 (73.2) | 0.730 |
| A patient with delirium is likely to be easily distracted  and/or have difficulty following a conversation (True) | 111 (94.1) | 39 (95.0) | 0.826 |
| Patients with delirium will often experience perceptual  disturbances (True) | 102 (86.4) | 38 (92.7) | 0.288 |
| Altered sleep/wake cycle may be a symptom of delirium  (True) | 107 (90.7) | 38 (92.7) | 0.696 |
