# Supplementary material for: A delirium prevalence audit and a pre and post evaluation of an interprofessional education intervention to increase staff knowledge about delirium in older adults
Source: BMC Nurs. 2021 Oct 19;20:202. doi: 10.1186/s12912-021-00692-2 (PMC8525041; doi:10.1186/s12912-021-00692-2)
Supplement: Supplementary file 3 — Additional file 3: Table C. Results for questions relating to risk of delirium [file 12912_2021_692_MOESM3_ESM.docx]

Table C. Results for questions relating to risk of delirium

|  | Pre - Correct  Answer n (%) | Post -  Correct answer n (%) | P-value |
| --- | --- | --- | --- |
| A patient having a repair of a fractured neck of femur  has the same risk of delirium as a patient having an elective hip replacement (False) | 34 (29.1) | 15 (36.6) | 0.370 |
| The risk of delirium increases with age (True) | 99 (84.6) | 35 (85.4) | 0.908 |
| A patient with impaired vision is at increased risk of  delirium (True) | 27 (22.9) | 11 (26.8) | 0.610 |
| The greater the number of medications a patient is  taking, the greater their risk of delirium (True) | 96 (81.4) | 35 (85.4) | 0.561 |
| A urinary catheter in situ reduces the risk of delirium  (False) | 96 (81.4) | 38 (92.7) | 0.086 |
| Gender has no effect on the development of delirium  (False) | 17 (14.5) | 14 (34.2) | **0.006** |
| Poor nutrition increases the risk of delirium (True) | 81 (68.6) | 29 (70.7) | 0.803 |
| Dementia is the greatest risk factor for delirium (True) | 58 (49.6) | 20 (48.8) | 0.930 |
| Males are more at risk for delirium than females (True) | 9 (7.6) | 3 (7.3) | 0.948 |
| Diabetes is a high risk factor for delirium (False) | 30 (25.6) | 14 (34.2) | 0.296 |
| Dehydration can be a risk factor for delirium (True) | 114 (96.6) | 39 (97.5) | 0.781 |
| Hearing impairment increases the risk of delirium (True) | 32 (27.4) | 14 (34.2) | 0.410 |
| Obesity is a risk factor for delirium (False) | 73 (61.9) | 26 (65.0) | 0.723 |
| A family history of dementia predisposes a patient to  delirium (False) | 42 (35.6) | 27 (65.9) | **0.001** |
