# Supplementary material for: A delirium prevalence audit and a pre and post evaluation of an interprofessional education intervention to increase staff knowledge about delirium in older adults
Source: BMC Nurs. 2021 Oct 19;20:202. doi: 10.1186/s12912-021-00692-2 (PMC8525041; doi:10.1186/s12912-021-00692-2)
Supplement: Supplementary file 4 — Additional file 4: Table D. Descriptive statistics of audit findings by year. *health record [file 12912_2021_692_MOESM4_ESM.docx]

Table D. Descriptive statistics of audit findings by year

| Demographics and Admission history | 2017 (n=246) | 2018 (n=149) |
| --- | --- | --- |
| Age, |  |  |
| 65-74 | 91 (37.0%) | 95 (63.8%) |
| 75-84 | 106 (43.1%) | 49 (32.9%) |
| >85 | 49 (19.9%) | 5 (3.4%) |
| Gender |  |  |
| Male | 115 (46.8%) | 77 (51.7%) |
| Female | 131 (53.2%) | 72 (48.3%) |
| Length of stay, |  |  |
| <2 days | 68 (27.8%) | - |
| 2-5 days | 89 (36.3%) | - |
| 5-10 days | 48 (19.6%) | - |
| <4 days | - | 73 (49.0%) |
| 4-10 days | - | 60 (40.3%) |
| >10 days | 40 (16.3%) | 16 (10.7%) |
| Admitting Team, |  |  |
| General Medicine | 149 (60.6%) | 83 (55.7%) |
| General Surgery | 42 (17.1%) | 30 (20.1%) |
| Orthopaedics | 1 (0.4%) | 1 (0.7%) |
| DRAC/PRACWA | 9 (3.7%) | 15 (10.1%) |
| Respiratory | 6 (2.4%) | 5 (3.4%) |
| Other | 39 (15.9%) | 15 (27.8%) |
| Cognitive Assessment Completed, |  |  |
| Yes | 21 (8.5%) | 64 (43.0%) |
| Assessment Tool Used, |  |  |
| HR* 601-0 Cognitive Impairment/Delirium Screening Tool | 0 | 58 (90.6%) |
| HR 758 OT Montreal Cognitive Assessment | 11 (61.1%) | 3 (4.7%) |
| HR 875 Aged care Mini Mental and GDS | 1 (5.6%) | 0 |
| Other | 6 (33.3%) | 3 (33.3%) |
| Previous History of dementia, |  |  |
| Yes | 28 (11.4%) | 11 (7.4%) |
| Diagnosis of Delirium, |  |  |
| Yes | 16 (6.5%) | 7 (4.7%) |
| Cause of Delirium Trigger Documented, |  |  |
| Medication | 3 (18.8%) | 1 (14.3%) |
| Sepsis | - | 2 (28.6%) |
| Not documented | 13 (81.2%) | 3 (42.8%) |
| Other | - | 1 (14.3%) |
| ICU/HDU Admission, |  |  |
| Yes | 7 (2.9%) | 8 (5.4%) |
| MET Calls |  |  |
| Yes | 8 (3.3%) | 6 (4.0%) |
| Falls this admission, |  |  |
| 1 | 6 (2.4%) | 1 (0.7%) |
| 2 | 3 (1.2%) | - |
| None | 237 (96.3%) | 148 (99.3%) |
| Code Black this Admission, |  |  |
| 1 | 1 (0.4%) | - |
| 2 | 1 (0.4%) | - |
| None | 244 (99.2%) | 149 (100.0%) |
| Sedation Medication for Agitation/Aggression |  |  |
| Antipsychotics | 3 (1.2%) | 2 (1.4%) |
| Benzodiazepines | 8 (3.3%) | - |
| Both | 2 (0.8%) | 1 (0.7%) |
| None | 233 (94.7%) | 144 (98.0%) |
| Medication for Agitation, |  |  |
| Yes | 13 (5.3%) | 2 (1.4%) |
| Required one-to-one companion, |  |  |
| Yes | 13 (5.3%) | 2 (1.3%) |
| Death, |  |  |
| Yes | 10 (4.1%) | 5 (3.4%) |
| Discharged home with higher level of care, |  |  |
| Yes, residential aged care | - | 6 (4.1%) |
| Yes, home with support | - | 10 (6.9%) |
| No, same as pre admission | - | 129 (89.0%) |

*health record
